# AI–Driven Multimodal Sensing for Early Detection of Health Disorders in Dairy Cows

**DOI:** 10.3390/ani16030411

**Published:** 2026-01-28

**Authors:** Agne Paulauskaite-Taraseviciene, Arnas Nakrosis, Judita Zymantiene, Vytautas Jurenas, Joris Vezys, Antanas Sederevicius, Romas Gruzauskas, Vaidas Oberauskas, Renata Japertiene, Algimantas Bubulis, Laura Kizauskiene, Ignas Silinskas, Juozas Zemaitis, Vytautas Ostasevicius

**Affiliations:** 1 Artificial Intelligence Excellence Centre, Kaunas University of Technology, 51423 Kaunas, Lithuania; 2 Department of Applied Informatics, Faculty of Informatics, Kaunas University of Technology, 51368 Kaunas, Lithuania; 3 Department of Anatomy and Physiology, Faculty of Veterinary Medicine, Lithuanian University of Health Sciences, 47181 Kaunas, Lithuania; 4 Institute of Mechatronics, Kaunas University of Technology, 51424 Kaunas, Lithuania; 5 Department of Mechanical Engineering, Mechanical Engineering and Design Faculty, Kaunas University of Technology, 51424 Kaunas, Lithuania; 6 Centre for Digestive Physiology and Pathology, Department of Anatomy and Physiology, Faculty of Veterinary Medicine, Lithuanian University of Health Sciences, 47181 Kaunas, Lithuania; 7 Department of Animal Breeding, Veterinary Academy, Lithuanian University of Health Sciences, 47181 Kaunas, Lithuania; 8 Department of Computer Sciences, Faculty of Informatics, Kaunas University of Technology, 51368 Kaunas, Lithuania

**Keywords:** dairy cow, mastitis, thermal imaging, artificial intelligence, computer vision, early prediction

## Abstract

Digital technologies that continuously quantify animal behavior, physiology, and production offer significant potential for the early identification of health and welfare disorders of dairy cows. In this study, a multimodal artificial intelligence (AI) framework is proposed for real-time health monitoring of dairy cows through the integration of physiological, behavioral, production, and thermal imaging data, targeting veterinarian-confirmed udder, leg, and hoof infections. Predictions are generated at the cow-day level by aggregating multimodal measurements collected during daily milking events. The dataset comprised 88 lactating cows, including veterinarian-confirmed udder, leg, and hoof infections grouped under a single ‘sick’ label. To prevent information leakage, model evaluation was performed using a cow-level data split, ensuring that data from the same animal did not appear in both training and testing sets. The system is designed to detect early deviations from normal health trajectories prior to the appearance of overt clinical symptoms. All measurements, with the exception of the intra-ruminal bolus sensor, were obtained non-invasively within a commercial dairy farm equipped with automated milking and monitoring infrastructure. A key novelty of this work is the simultaneous integration of data from three independent sources: an automated milking system, a thermal imaging camera, and an intra-ruminal bolus sensor. A hybrid deep learning architecture is introduced that combines the core components of established models, including U-Net, O-Net, and ResNet, to exploit their complementary strengths for the analysis of dairy cow health states. The proposed multimodal approach achieved an overall accuracy of 91.62% and an AUC of 0.94 and improved classification performance by up to 3% compared with single-modality models, demonstrating enhanced robustness and sensitivity to early-stage disease.

## Simple Summary

This manuscript presents a multi-modal artificial intelligence framework for health status and welfare detection of dairy cows. Data from the milking system, internal sensing devices, and thermal cameras were jointly analyzed to enable comprehensive health monitoring. All measurements, except the internal sensor, are collected in a contactless manner, avoiding direct physical interaction with the animals. By linking changes in production and physiological data with visual temperature patterns, the system can identify health warning signs of disease more reliably than when using a single data source. The system incorporates a novel hybrid deep learning architecture that unifies the backbone structures of U-Net, O-Net, and ResNet, facilitating multi-scale feature learning for accurate analysis of dairy cow health conditions. This combined approach demonstrates improved discrimination performance in retrospective analysis and indicates potential for earlier identification of health-related deviations, which may support the development of decision-support tools for dairy cow health management.

## 1. Introduction

Ensuring high standards of welfare on dairy farms is essential for maintaining animal health, productivity, and overall farm sustainability. Welfare Quality (WQ) assessment protocols provide a structured framework for evaluating animal well-being, particularly through principles related to good health, such as the absence of disease, injuries, and prolonged discomfort. In this study, these principles are operationalized through continuous monitoring of mastitis-related udder inflammation, hoof- and leg-associated locomotor disorders, and associated changes in production and physiological indicators, enabling early detection of health and welfare impairments.

In recent years, rapid advances in artificial intelligence (AI) and sensor technologies have expanded the possibilities for objective evaluation of welfare. Artificial intelligence technologies, originally advanced in human precision medicine, are now widely applied to animal health, epidemiology, and disease surveillance, enhancing predictive accuracy and supporting faster, evidence-based decisions [1,2]. Most recent research focuses on multimodal AI, an emerging field that integrates heterogeneous data sources such as medical imaging, clinical information (e.g., laboratory reports) [3,4], behavioral video [5,6], and audio signals [7]. By fusing these modalities, AI enables a more comprehensive and data-driven approach to veterinary diagnostics, improving precision and efficiency. However, in contrast to human medicine, veterinary diagnostics must address species-specific physiology, variable behavioral patterns, and the lack of verbal symptom reporting [8], making multimodal data integration both challenging and essential.

Since diagnostic signs vary widely across species, multimodal systems must learn to interpret physiological signals, movement dynamics, and acoustic patterns in the context of each animal’s biology and behavior. This requires flexible architectures capable of handling high inter-species variability while preserving sensitivity to subtle, early-stage disease indicators [9,10]. In addition, animals often mask signs of illness, exhibit stress-induced behavioral changes, or demonstrate atypical movement patterns in farm environments, making it even more challenging to extract meaningful insights from video or sensor data [4,9]. Therefore, multimodal AI systems in veterinary medicine must not only integrate diverse data streams but also adapt to a heterogeneous biological base, environmental influences, and species-dependent disease patterns. These complexities underline the critical importance of robust data fusion strategies and domain-specific model design when developing AI solutions for comprehensive animal health monitoring.

These challenges are particularly evident in dairy cattle, where complex health disorders such as mastitis [11], lameness [12,13], and heat stress [14], as well as biological/reproductive status [15,16], require continuous, multimodal monitoring. As a result, cows have become a central focus for AI-driven welfare assessment, with recent advances demonstrating strong potential for early detection and intervention [17]. Recent studies emphasize the application of IoT and machine learning systems to monitor cattle health by capturing sensor-based data on feed and fluid intake [18], activity levels, and body condition [19]. However, even now, the majority of these technologies rely on collars, leg bands, rumen boluses, or other wearable devices that may influence the animal’s comfort, induce stress, or interfere with natural behavior. Their performance also depends heavily on device durability, battery life, environmental conditions, and farmer compliance, creating substantial barriers to long-term, large-scale implementation.

Given the limitations of conventional physical sensors, there is a growing need to develop advanced AI-based monitoring frameworks that emphasize contactless sensing while selectively incorporating minimally invasive technologies when justified. In this context, contactless modalities such as visual and thermal imaging enable continuous external monitoring with minimal animal disturbance, while minimally invasive internal sensors can provide complementary physiological information. This combined approach reflects a deliberate welfare—feasibility trade-off commonly adopted in precision livestock farming, balancing animal comfort with the need for reliable health indicators.

In this context, our study aims to leverage data from intra-ruminal bolus sensors and the DeLeval DelPro system, which together provide continuous measurements of core physiological parameters such as internal body temperature, activity, rumination behavior, water intake, and production metrics. These internal sensor streams will be complemented by thermal imaging data acquired through automated heat cameras installed in the milking station, enabling contactless monitoring of udder and limb temperature patterns associated with inflammation. By integrating these physiological, thermal, and environmental signals into a unified multimodal AI framework, we seek to identify early deviations from normal health trajectories and detect early-stage health disorders, specifically mastitis and infections affecting the udder, hoof, and leg. In this study, “early” disease detection is defined operationally as the identification of abnormal physiological, production, or thermal patterns during the incubation period preceding veterinarian-confirmed diagnosis and treatment. Based on veterinary records, this period was defined as up to 20 days before clinical intervention, allowing the model to target subclinical and preclinical stages of disease. This approach offers the potential for earlier interventions, improved welfare outcomes, and more efficient herd management compared with traditional single-modality or wearable-only monitoring systems.

## 2. Related Works

On large-scale dairy farms, where limited staff availability limits regular physical observation of individual animals, automated animal health monitoring systems have emerged as a practical and necessary approach in modern animal husbandry. In this context, the adoption of AI, sensor technologies, and Internet of Things (IoT) solutions in dairy farming and livestock export has been increasingly explored as a means to enhance animal welfare, operational efficiency, and long-term sustainability. Across the literature review, these technologies are generally presented as enabling continuous monitoring, data-driven decision making, and more transparent livestock management processes. Despite their conceptual appeal, the practical benefits of such systems remain ambiguous, and significant technical, data-related, and ethical challenges still limit their large-scale deployment [6,20].

As an initial and significant step toward the digitalization of cow-related parameters, a range of commercial precision livestock farming systems (such as Bolus, DeLaval herd management solutions, Lely Horizon, Afimilk, CowManager, Nedap, SCR Heatime, SenseHub, etc.) have been deployed to enable continuous monitoring of physiological, behavioral, and production-related indicators in dairy cattle. Invasive and wearable sensors deployed on or within dairy cows enable continuous monitoring of a wide range of parameters, including feeding and rumination behavior, rumen pH, body temperature, lying behavior, physical activity levels, and animal positioning or spatial distribution, among others [21]. Detailed reviews have shown that the accuracy of parameters generated by commercially available sensor systems can vary substantially, even among systems from the same manufacturer, with the underlying causes of these differences remaining unclear [22]. And very often, it is concluded that significant results in this field can only be achieved if modern computing tools and advanced data analysis tools are used at all levels [23,24].

**Figure 1:**
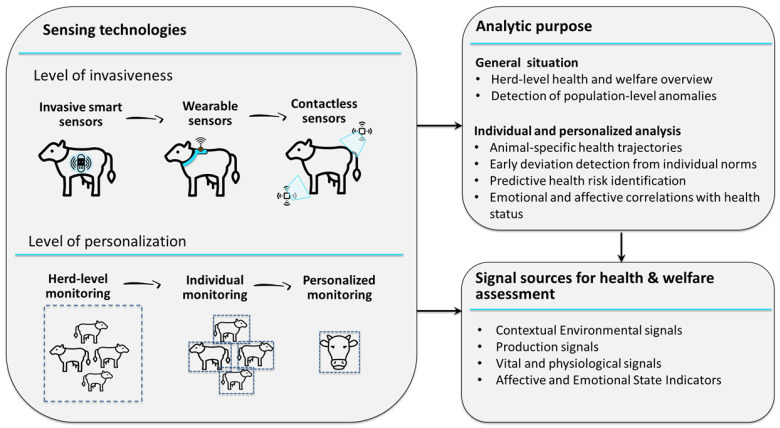
AI-enabled livestock monitoring framework integrating sensing technologies and analytical levels.

Nonetheless, the automated collection of health measurements in livestock is becoming increasingly important for both researchers and practitioners, as the ability to acquire real-time data non-invasively enables deeper insights into animal behavior and physiology and supports more informed and timely animal management decisions [25]. Although current solutions, particularly for cattle, often rely on wearable devices [26,27] or a combination of invasive, wearable, and contact-based sensing technologies, the long-term objective is to develop fully contactless monitoring systems. Continuous collection of physiological and behavioral indicators allows for early detection of deviations from the norm, which is essential for timely intervention before clinical symptoms appear. In parallel, integrated streams of physiological, behavioral, and environmental data form the basis for AI models to learn animal-specific baseline patterns and to identify subtle anomalies that may precede observable changes in activity [28], feeding behavior, rumination [23], or thermal responses [29]. Recent AI-based approaches have combined deep learning object detection models with customized tracking and identification algorithms to achieve high-accuracy cows detection, automated monitoring of individual animal behavior [30,31], including facial tracking recognition, and those patterns correlate with a health status [32,33]. Below (see Figure 1) is provided a conceptual scheme of AI-enabled possibilities for data-driven livestock monitoring systems, illustrating alternative sensing modalities with varying degrees of invasiveness, multiple levels of personalization in decision-making, and the concurrent integration of environmental, production, physiological, behavioral, and emerging emotional indicators for comprehensive assessment of animal health and welfare.

While a range of diseases can be detected through the smart monitoring systems, mastitis remains one of the most significant health challenges in dairy cattle. Mastitis has been extensively studied as one of the most prevalent and economically burdensome diseases in dairy cattle, due to its direct impact on milk quality, animal welfare, and antimicrobial usage [34,35]. Several studies have demonstrated that mastitis can be detected or predicted using diverse data sources and analytical approaches [36]. In particular, computer vision–based methods have been proposed to enable early, non-invasive mastitis detection [37], leveraging thermal imaging [38,39] and knowledge-transfer [40] strategies to distinguish between healthy and mastitis-affected cows with high classification accuracy. In most studies, mastitis is typically formulated as a binary [36] or three-class classification problem [41], where cows are categorized as healthy, affected by subclinical mastitis, or affected by clinical mastitis. AI-based models are used to learn discriminative patterns from sensor, production, imaging, or thermal data. Such approaches can significantly reduce the reliance on manual inspection. This typically requires integrating multimodal data sources. However, reliable early prediction remains challenging due to the complex and multifactorial nature of mastitis, substantial inter-animal variability, environmental influences, and sensor noise. In addition, the availability of high-quality ground-truth labels (i.e., consistent veterinary diagnoses, detailed clinical records, accurate annotations, etc.) is often limited, resulting in scarce, delayed, or poorly labeled datasets. This lack of reliable reference labels significantly constrains the generalization of AI-based models, making robust early mastitis prediction an ongoing open research problem.

Lameness represents another of the most frequently observed health disorders in dairy cattle and constitutes a major source of animal welfare impairment in modern dairy farming systems [42]. Lameness is common and painful in dairy cattle [43]. Still, mild cases are frequently overlooked because farmers often lack time, training, clear treatment guidelines, and perceive limited economic benefit, highlighting the need for better early-detection tools and clearer best management practices to support timely identification and treatment [44]. Consequently, a growing body of research has investigated AI, and particularly computer vision–based approaches for the early detection and assessment of lameness, utilizing gait analysis [45,46], posture evaluation [47], activity patterns, and locomotion asymmetry as key indicators [48]. Kinematic and kinetic technologies provide objective and detailed locomotion measurements for early lameness detection, but their on-farm adoption remains limited due to methodological inconsistencies and the need for further technical standardization [49].

Collectively, these studies demonstrate that substantial progress has been made in the detection and prediction of mastitis and locomotion-related disorders using artificial intelligence, sensor technologies, and advanced data analytics. Nevertheless, persistent challenges related to multimodal data integration, robustness across heterogeneous farm environments, scalability to large herds, limited availability of high-quality reference labels, and the explainability of AI-driven decisions continue to constrain widespread adoption in real-world settings. These limitations underscore the need for holistic, multimodal, and welfare-centered monitoring frameworks that combine reliable sensing, advanced AI/ML methodologies, and domain knowledge to enable early, accurate, and actionable health assessment in dairy cattle.

## 3. Materials and Methods

This study presents a multimodal artificial intelligence framework for real-time health monitoring in dairy cows, integrating physiological, production, and thermal imaging data into a unified diagnostic system. The framework was developed to detect early deviations indicative of emerging disease, including conditions that may not yet manifest visible clinical signs. All measurements were obtained using non-invasive technologies installed within a commercial dairy operation equipped with automated monitoring and milking systems, except for the intra-ruminal bolus sensor, which requires oral administration and is therefore invasive.

The investigation was carried out on a single dairy farm in Lithuania between 1 January and 20 October 2025. The study population consisted of 88 lactating cows housed in free-stall barns. All cows were maintained under standardized farm management conditions. Animals were fed a total mixed ration (TMR) formulated according to the farm’s nutritional program for lactating dairy cows and had ad libitum access to drinking water throughout the study period. Veterinary examinations were performed at regular intervals, and all health- and production-related information was continuously documented in a centralized digital herd management system. Cows selected for the study met the following inclusion criteria:

- At least one completed lactation cycle at study beginning;
- Continuous enrollment in the automated milking system;
- No chronic or terminal disease diagnosis prior to study.

**Figure 2:**
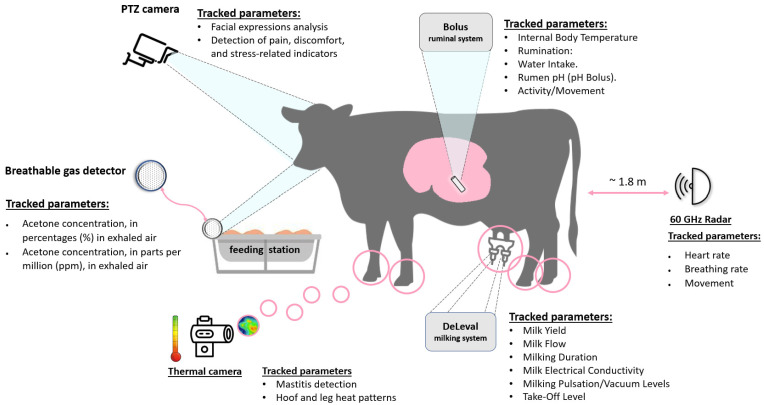
Overview diagram of the cow health-monitoring systems.

For non-invasive cow health assessment, these on-farm sensor systems (see Figure 2) were used:

- A DeLaval milking system (DeLaval Inc., Tumba, Sweden) was used to milk 88 cows (~2–3 times daily) in a parlour system.
- At the start of the study, each of the 88 cows was equipped with an orally administered SmaXtec Classic Bolus (Model: SCB-G5, SmaXtec Animal Care GmbH, Graz, Austria).
- A thermal camera (Model: Topdon TC001, Topdon Technology Co., Ltd., Shenzhen, China) was installed in front of the milking robot at approximately 40 cm above ground level, oriented toward the udder and lower limb region of the cow. To protect the device from dust, humidity, and mechanical damage in the aggressive farm environment, the camera was housed in a sealed protective enclosure. Prior to deployment, the camera was calibrated using the manufacturer’s built-in software calibration procedure to ensure temperature measurement accuracy.
- Hikvision mini PTZ camera (Model: DS-2DE2A404IW-DE3, Hangzhou Hikvision Digital Technology Co., Ltd., Hangzhou, China) was used for observing cow welfare by detecting head and ear positions. Animal behavior is a critical indicator reflecting physical and psychological well-being. Behavioral alterations can signal stress, pain, discomfort, illness, or herd social dynamics. Head and ear position, while subtle, are highly informative behavioral cues that reveal comfort, stress, pain, or early disease symptoms (e.g., drooped ears/head, unnatural tilt, unusual movements).
- DFRobot (SEN 0632) C1001 60 GHz mmWave Human Detection Sensor (Zhiwei Robotics Corp., Shanghai, China) was used for heart rate and breath rate measurements. Animal pulse and breath rate changes are vital physiological indicators signaling stress, pain, fever, infections, or other health disorders. Elevated heart rate may indicate fever, pain, or fear; irregular rhythm could point to cardiac issues. Traditional pulse measurement is challenging in large farms, inducing animal stress and demanding continuous staff involvement. To address these limitations, a non-invasive and continuous monitoring approach was explored using non-contact mmWave radar sensing. The 60 GHz mmWave radar sensor was first evaluated under controlled laboratory conditions, where stable and accurate heart and respiration rate measurements were obtained, and was subsequently deployed on the farm. The sensor was mounted inside a sealed enclosure at a height of approximately 1.10 m above ground level and positioned about 2.5 m from the cow in the milking station, oriented toward the animal’s thoracic region to enable remote physiological monitoring without direct skin contact.
- MQ138 gas sensor (Zhengzhou Winsen Electronics Technology Co., Ltd., Zhengzhou, China) was used to detect acetone in the exhaled air. It is an innovative acidosis diagnosis method via exhaled breath analysis. Acidosis, especially subclinical, is a widespread issue in dairy cows, arising from improper nutrition (e.g., excessive rapidly fermentable carbohydrates) and impaired digestive function [50,51]. It causes rumen dysfunction, reducing milk productivity, degrading quality (decreased fat/protein), and increasing risk of lameness, mastitis, or laminitis [50]. Traditional acidosis detection methods are invasive, demanding specialized equipment and personnel, and impractical for regular application across large animal populations. In this study, the MQ138 gas sensor was employed as a low-cost, pilot device for preliminary feasibility evaluation of breath-based acetone detection; no formal laboratory calibration was performed, and the sensor was therefore used solely for exploratory assessment, with its practical limitations discussed accordingly in Section 6.

Although multiple sensing technologies were deployed during the study, only production data from the automated milking system, intra-ruminal bolus measurements, and thermal imaging were used in the final disease prediction model. The remaining sensors were evaluated for exploratory feasibility and were not included in model training or evaluation.

## 4. Data

Data acquisition is the first stage of the pipeline, encompassing a distributed sensor infrastructure that captures multimodal data. Three complementary sensing modules are deployed:

(1) Thermal imaging module. A thermal camera is installed in the milking station to acquire thermal images of each cow. Importantly, the image is captured at the beginning of the milking process. This timing is selected to ensure standardization, as the cow is stationary, positioned consistently at this moment and is not warmed by apparatus and udder cleaning process. The thermal image is used for physiological monitoring, by detecting localized heat signatures indicative of inflammation, mastitis, or systemic illness.
(2) Milking station sensors. The milking station functions both as an identification hub and as a rich source of physiological and production data. Each cow is identified upon entry via radio-frequency identification (RFID). During the milking session, the station records milk yield as the total volume produced per session and measures milk flow rate from each quarter of the udder to detect asymmetries or irregularities associated with udder health problems. In addition, the station captures milk quality indicators, including the presence of blood in milk, electrical conductivity, and other anomalies that may reflect mastitis or other disorders. These measurements provide valuable insights not only into productivity but also into udder physiology and potential subclinical infections.
(3) Bolus module (Intra-ruminal sensor). The bolus module, inserted into the cow’s stomach, continuously measures internal body temperature and digestive parameters, including rumination time, water intake, and activity-based indicators associated with digestive and metabolic function. These data streams capture essential physiological signals related to metabolic function, thermoregulation, and feed intake behavior. Such indicators are crucial for early detection of systemic health problems, including fever and digestive dysfunctions. Although the bolus sensor does not directly measure rumen pH, these parameters may provide indirect indications of metabolic imbalance.

All sensor readings are timestamped and synchronized to ensure alignment across modalities. Numerical data from the intra-ruminal bolus sensor, recorded at 10-min intervals, and production data from the milking system were aligned to daily health labels by aggregating values within each calendar day at the cow level.

Different aggregation functions were applied depending on the physiological meaning of each variable. Continuous state variables such as internal temperature, average milk flow, and rumination-related parameters were aggregated using daily mean values. Cumulative production variables, including daily milk yield and milk yield per udder quarter, were aggregated using daily sums. Peak-related indicators, such as maximum milk flow per udder quarter, were aggregated using daily maximum values.

During preprocessing, only measurements affected by evident sensor or system malfunctions (e.g., missing values, duplicated timestamps, or clearly erroneous machine outputs) were removed. The milking station identification guarantees that every data record is associated with the correct individual cow, forming the foundation of the monitoring framework.

Data collection relied on multiple sensors integrated into the farm’s automated monitoring infrastructure, as well as operational farm management parameters. Table 1 summarizes all data sources, including their sampling frequency, duration of data collection, and the specific parameters recorded. Each sensor modality contributed complementary information regarding the cow’s physiological condition, behavior, and productivity, enabling the development of a multimodal dataset suitable for health status prediction. Numerical data collected over the extended January–October period were used exclusively for data exploration, feature engineering, and identification of informative attributes. For model training and evaluation, numerical features were restricted to the same 5-day period during which thermal images were collected, ensuring temporal consistency across modalities in the multimodal experiments.

Raw data collected from sensors are stored in cow-specific databases, which support the creation of continuous, individualized health profiles. The system employs a structured aggregation strategy to transform raw measurements into meaningful, temporally consistent features:

- Milk yield: Aggregated into daily totals, enabling detection of changes in productivity trends over time.
- Milk flow rate: Profiled per udder quarter, preserving information about asymmetry and temporal flow dynamics. Such granularity enhances the sensitivity of the system to early signs of udder infections.
- Milk quality indicators: Parameters such as the presence of blood or changes in conductivity are stored as event-level features and aggregated to reflect daily frequencies or severities.
- Bolus module data: Reduced to daily mean, variance, and anomaly indicators from continuous temperature and digestive activity recordings. These features reflect metabolic stability and thermal homeostasis.
- Thermal images: Stored and linked to each milking event over five consecutive days (three images per milking session), ensuring consistency across days and allowing for robust image-based feature extraction.

Outliers that were physiologically plausible for a given cow, based on individual baseline characteristics and veterinary knowledge, were retained, as such deviations may represent early indicators of health disorders rather than noise. Missing bolus intervals due to short-term sensor dropouts were excluded from aggregation; no interpolation or forward-filling was applied. After aggregation and data augmentation, each cow was represented by one numerical feature vector per day, resulting in 293 numerical records per cow over the study period. Numerical features were normalized using min–max scaling on a per-cow basis to account for inter-animal variability while preserving biologically meaningful deviations.

Thermal images were collected during milking sessions over a limited five-day period. Because cows typically visited the milking station multiple times per day, multiple thermal images were acquired per cow per day, whereas numerical sensor data were available as a single aggregated cow-day record. For multimodal model training, each thermal image was paired with the same corresponding daily numerical feature vector of the respective cow. This approach preserves image-level variability across milking events while maintaining consistency with cow-day–level numerical features and health labels.

This aggregation phase reduces noise while preserving the richness of multimodal information. It also ensures comparability across animals and across time, enabling effective temporal modeling.

Due to the reason that cows’ health parameters are analyzed to predict possible cases of mastitis, the majority of parameters are directly connected to milk production (see Table 2). To annotate the dataset, the veterinarian’s indication of illness is used. On those dates, when veterinarians mark that the cow was diagnosed with mastitis or received the dose of medicine, it is marked in the parameter “is sick” as 2. However, cases of mastitis are not detected instantly, and health parameters can change before the diagnosis. Those 20 incubation days are marked in the column “is sick” as 1. Otherwise, if the cow is not diagnosed with mastitis or is not in the incubation period, it is marked as healthy (0).

Thermal images were captured using a fixed-position infrared camera (specifications presented in Table 3 installed in the milking stall oriented toward the udder region to ensure repeatable positioning.

**Figure 3:**
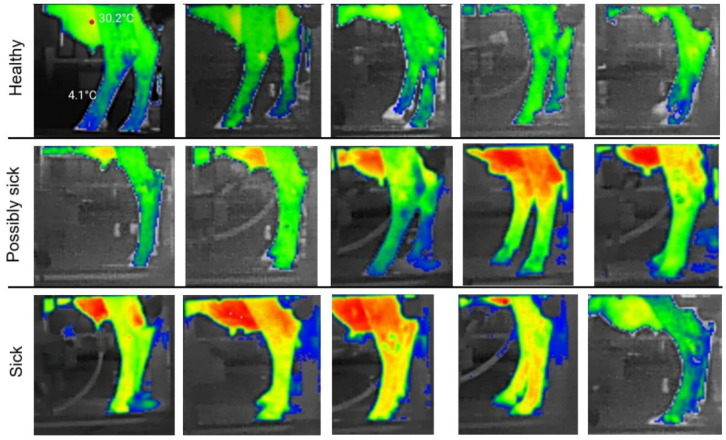
Examples of thermal images of healthy, possibly sick, and sick cows.

To maintain consistency, thermal frames were captured before udder cleaning or milk contact, minimizing heat artifacts caused by friction, cleaning fluids, or mechanical stimulation. Figure 3 represents thermal images from healthy and sick cows.

For model development and evaluation, the classification task was formulated as a binary problem. Only two classes, healthy and sick, were used for training and testing, where the ‘sick’ class included veterinarian-confirmed udder, leg, and hoof infections. The ‘possibly sick’ category was not used for model training and served solely as a visual aid in Figure 3.

Cows were classified as sick only when a veterinarian confirmed an infection localized in the udder, hoof, or leg. Other health disorders or systemic illnesses were excluded from the dataset and not considered in the analysis.

In Figure 3, the thermal image shows a pronounced temperature increase, highlighted in red, characteristic of an advanced udder infection. In contrast, thermal differences associated with early-stage infections are often subtle and difficult to distinguish visually. Therefore, reliable early prediction requires multimodal data analysis rather than reliance on visual thermal cues alone.

The thermal image dataset exhibited a pronounced class imbalance, with 480 images available for healthy cows and 166 images for infected cows in the original dataset. To mitigate this imbalance, data augmentation was applied to the thermal images, increasing the total number of images to 1790 (960 healthy and 830 infected). The dataset was first divided into training, validation, and test sets at the cow level, and augmentation was then performed separately within each split to avoid information leakage across datasets. Data augmentation included random rotations, horizontal flipping, scaling, and slight intensity variations applied to the thermal images. Images of healthy cows were augmented only twice, while images of cows labeled as sick were augmented five times. This approach allowed the dataset to be balanced without excessively oversampling the healthy class, while ensuring that the minority class (sick cows) had sufficient representation for model training and improved generalization performance.

### Proposed Model

**Figure 4:**
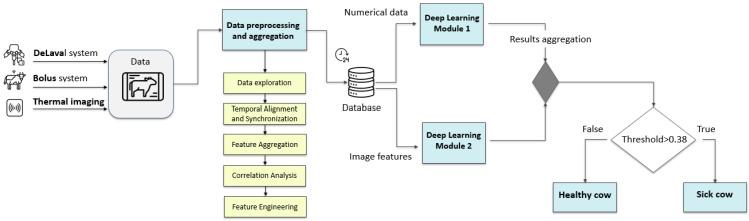
Schematic diagram of the proposed decision-making algorithm.

Figure 4 illustrates the complete workflow of the multimodal cow health-monitoring system, from data acquisition through health classification. Although data were available from six independent sources, the present analysis focused on three (milking station data, thermal imaging, and ruminal bolus measurements) selected for their greater informativeness and relevance to health status assessment and early disease detection. All incoming data streams are directed into a preprocessing and aggregation unit. In this step, raw inputs are cleaned, synchronized by timestamp and cow identification, and transformed into structured, daily representations. Once processed, the numerical data and extracted image features are stored in a central database, ensuring consistent traceability and temporal alignment across modalities.

From the database, numerical data are routed to a deep learning module that learns temporal and physiological patterns from historical behavior and production trends. In parallel, the thermal images are processed using an O-Net–inspired [52,53] architecture equipped with a ResNet [54] encoder. This model extracts high-level spatial features from the udder and leg thermal profiles, enabling the detection of subtle temperature asymmetries that may indicate localized inflammation.

Multimodal fusion is performed at the decision level by averaging the class probability outputs obtained from the numerical and image branches. The final health prediction is determined based on this averaged probability score. This fusion strategy enables complementary integration of numerical and visual information while maintaining independent modality-specific learning. The final decision is based on a predefined threshold: if the predicted probability exceeds 0.38, the cow is classified as sick; otherwise, it is marked as healthy. Through this integrated approach, the system can detect both advanced and early-stage infections using complementary sensor information rather than relying on a single data type.

The proposed image processing network is built upon a ResNet-50–derived encoder composed of convolutional layers with residual skip connections. This design facilitates the learning of deep hierarchical feature representations by promoting stable gradient propagation and mitigating the degradation issues commonly encountered in very deep architectures. The encoder generates five successive feature stages (B1–B5), where early stages preserve fine-grained spatial information and deeper stages capture increasingly abstract semantic representations.

Each encoder stage is subsequently processed using a Pyramid Pooling Module (PPM), adapted from the O-Net family [52,53] of architectures. The PPM performs pooling operations at multiple spatial scales, including global, half-scale, and quarter-scale pooling, followed by upsampling to a common spatial resolution. This multi-scale aggregation enables the integration of local texture details with global contextual information, which is particularly important when object appearance, scale, or orientation varies across samples. The pooled outputs from all pyramid levels and all encoder stages are concatenated to form a unified multi-scale feature representation that combines complementary spatial and semantic cues.

Rather than applying feature flattening, the aggregated multi-scale tensor is compressed using GlobalAveragePooling2D, yielding a compact descriptor in which each channel corresponds to a learned global semantic feature. This descriptor is then passed to a lightweight classification head composed of fully connected layers with dropout regularization, producing a single probability value through a sigmoid activation function for binary classification.

Compared with standard architectures, the proposed hybrid model occupies an intermediate position. Conventional ResNet-based classifiers rely on global pooling but lack explicit multi-scale feature integration. U-Net architectures, which are used in this study only as comparative baselines, reconstruct high-resolution feature maps for dense prediction tasks, such as segmentation, but are not optimized for compact classification embeddings. O-Net models capture multi-scale contextual relationships through pyramid pooling but are primarily designed for pixel-wise output. Inspired by these complementary design principles, the proposed architecture integrates residual encoding, pyramid pooling–based contextual modeling, and global average pooling for classification. This design enables strong contextual awareness while maintaining the computational efficiency and generalization capability of modern image classification networks.

**Figure 5:**
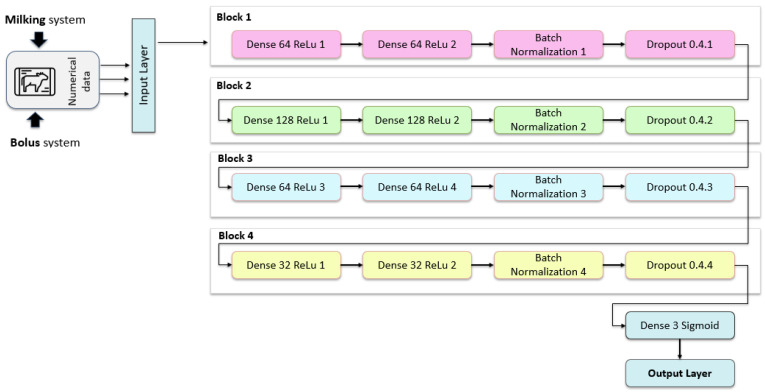
Schematic diagram of proposed model architecture for numerical data processing.

The diagram in Figure 5 illustrates the architecture of the numerical data processing branch used in the proposed multimodal framework. Numerical inputs derived from farm sensors and management systems are first passed through an input layer, where features are normalized and prepared for sequential processing. The network is structured as a series of fully connected blocks, each designed to progressively extract higher-level representations while controlling overfitting and ensuring stable convergence.

The first two blocks consist of dense layers with 64 and 128 neurons, respectively, followed by ReLU activation functions. These blocks are responsible for learning complex nonlinear relationships among physiological and production-related features. Batch normalization layers are applied after each block to stabilize learning and mitigate internal covariate shift, while dropout regularization helps prevent overfitting and improve generalization. The deeper blocks reduce the dimensionality to 64 and then 32 neurons, encouraging the network to compress information into a compact latent representation that captures the most discriminative numerical patterns associated with health status.

**Figure 6:**
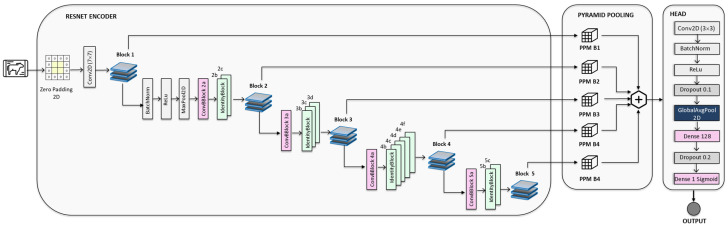
Schematic diagram of the proposed model architecture for image data processing.

Finally, the processed numerical features are passed to an output layer consisting of a dense layer with a sigmoid activation function, producing a probabilistic prediction. This output is later combined with image-derived features in the multimodal fusion stage. The layered design of the numerical branch allows the model to learn both short-term fluctuations and long-term trends in sensor data, making it well-suited for detecting subtle physiological deviations that may precede visible clinical symptoms. For image processing, a developed hybrid model architecture is presented below (see Figure 6).

Early infection detection in thermal imagery presents specific challenges, as initial inflammatory processes are typically manifested by subtle, spatially diffused temperature variations rather than distinct or well-localized visual patterns. To address these challenges, the proposed architecture integrates a ResNet-50–based encoder with pyramid pooling layers, enabling the extraction of deep discriminative features while preserving both local thermal details and global contextual information. The residual learning framework supports stable training of a deep network and enhances sensitivity to nuanced temperature gradients and spatial asymmetries that are indicative of early pathological changes.

The incorporation of pyramid pooling layers further strengthens the model’s representational capacity by aggregating features across multiple spatial scales. This multi-scale contextual modeling allows the network to jointly analyze fine-grained thermal irregularities and broader anatomical temperature distributions, thereby improving robustness to variability in infection size, anatomical location, and animal posture. This level of contextual awareness is especially critical in thermal imaging applications, as early pathological changes are often expressed as diffused thermal imbalances rather than distinct hotspots.

Conventional architectures such as U-Net and You Only Look Once (YOLO) are less appropriate for addressing the specific requirements of this task. U-Net is primarily optimized for segmentation problems involving distinct structural boundaries and does not explicitly model global context, while YOLO is designed for object detection scenarios that assume well-defined target instances. Because early-stage infections do not correspond to discrete objects or clearly separable regions, these models may struggle to generalize reliably. In contrast, the proposed architecture is explicitly tailored to capture subtle, context-dependent thermal patterns, making it more appropriate for early infection screening in real-world dairy farm environments.

**Figure 7:**
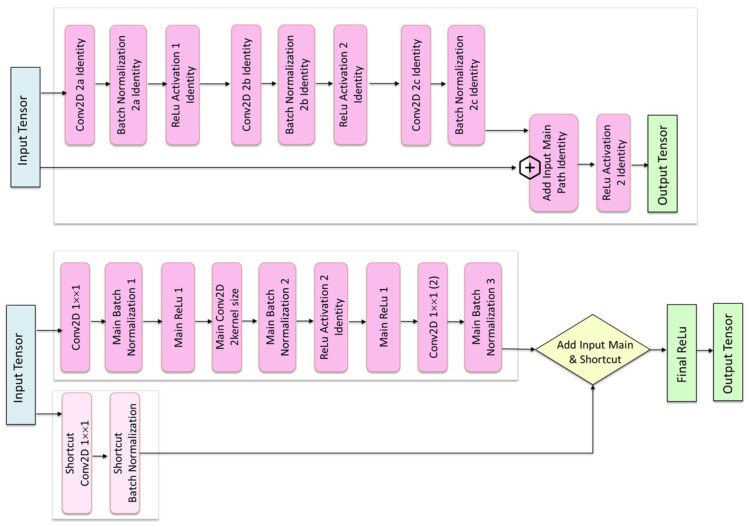
Schematic diagram of identity and convolution blocks.

The image depicts the two core components of the ResNet architecture: the identity block (top) and the convolutional block (bottom) (see Figure 7. Schematic diagram of identity and convolution blocks). In the identity block, the input tensor is processed through a sequence of convolution, batch normalization, and ReLU layers, while a direct shortcut connection adds the original input to the processed output. Because the input and output dimensions are identical, no transformation is applied to the shortcut path. In the convolutional block, used when spatial resolution or channel depth changes, the main convolutional path is accompanied by a shortcut path that includes its own convolution and batch normalization to match dimensions before the element-wise addition. In both cases, the final ReLU activation produces the output tensor. The figure illustrates how residual connections enable effective training of deep networks by facilitating gradient flow and learning residual mappings rather than direct representations.

## 5. Experimental Results

### 5.1. Numerical Data Analysis

**Figure 8:**
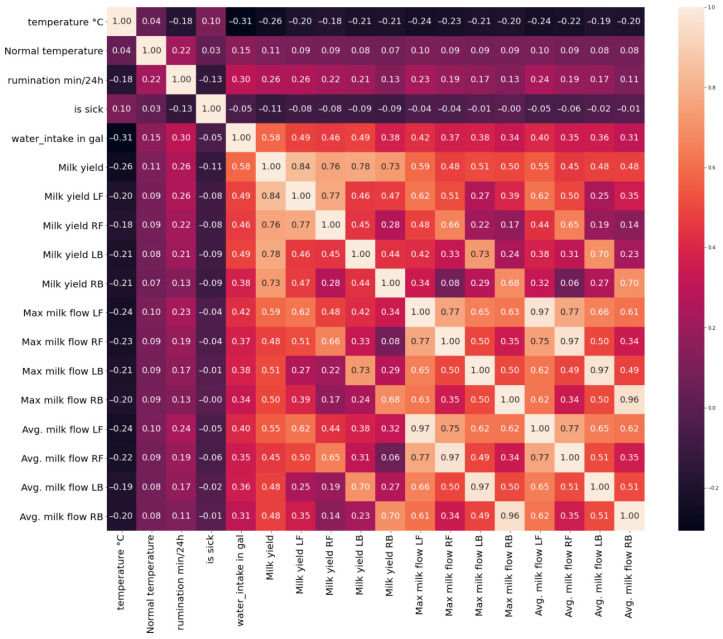
The correlation matrix of 88 cows.

To analyze the impact of changes in health parameters on illness status, the Pearson correlation (1) is calculated. In the image below (see Figure 8), the correlation for the dataset containing 88 cows is presented. While correlations between several parameters are observable, no strong linear correlation with the binary “is sick” variable is identified. This absence of clear correlation can be attributed to substantial inter-animal variability in physiological and behavioral responses to disease. Similar to humans, individual cows may exhibit different responses to the same health condition, indicating that population-level correlation analysis is insufficient and that individualized, cow-specific analysis is required for reliable disease detection.

(1) $$\rho_{X,Y}=\frac{cov(X,Y)}{\sigma_{X}\sigma_{Y}},$$

where $\rho_{X,Y}$—Pearson correlation, cov(X,Y)—covariance between *X* and *Y* attributes, $\sigma_{X}$—standard deviation of *X*, $\sigma_{Y}$—standard deviation of *Y*.

**Figure 9:**
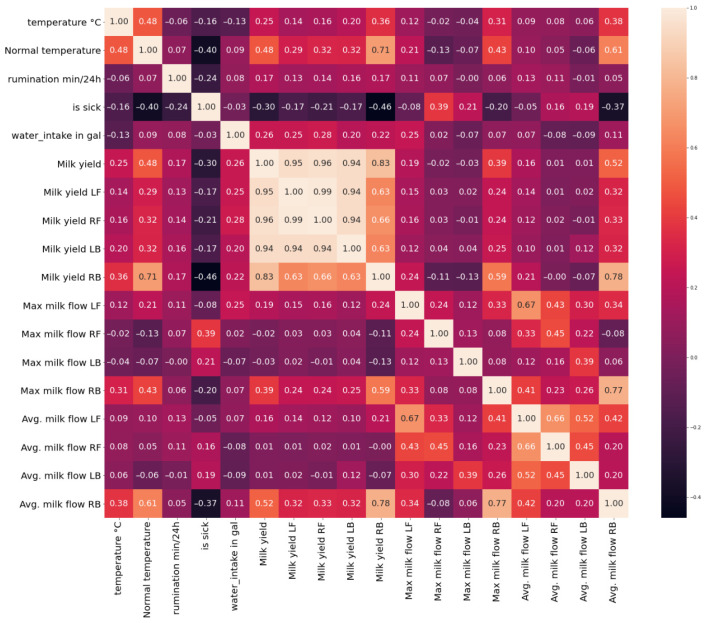
Correlation matrix for one sick cow.

At the individual-animal level, no strong linear correlation is observed between most health-related parameters and the sickness indicator. This observation highlights the limitations of simple correlation analysis for disease detection at the single-cow scale, particularly when productivity-related parameters are considered. Importantly, in this study, correlation analysis was not used to assess the predictive usefulness of individual features, but rather as an exploratory preprocessing step to identify highly collinear variables and reduce multicollinearity prior to model development, thereby supporting dimensionality reduction and numerical stability. Nevertheless, several variables exhibit comparatively higher correlations with the sickness and can therefore be considered informative. One example of an individual cow correlation matrix is presented below (Figure 9). For this cow, only weak to moderate linear correlations are observed with the sickness indicator. The strongest negative correlation is found for milk yield in the right back (RB) quarter ($\rho_{X,Y}\;\approx \;-0.46$), followed by normal body temperature ($\rho_{X,Y}\;\approx \;-0.40$) and average milk flow in the right back quarter ($\rho_{X,Y}\;\approx \;-0.37$). Rumination time also shows a weak negative correlation with the sickness label ($\rho_{X,Y}\;\approx \;-0.24$). All remaining parameters exhibit very low correlations with sickness ($\left|\rho_{X,Y}\right|<0.2$), indicating that no single variable is strongly linearly associated with disease status at the individual-cow level.

**Figure 10:**
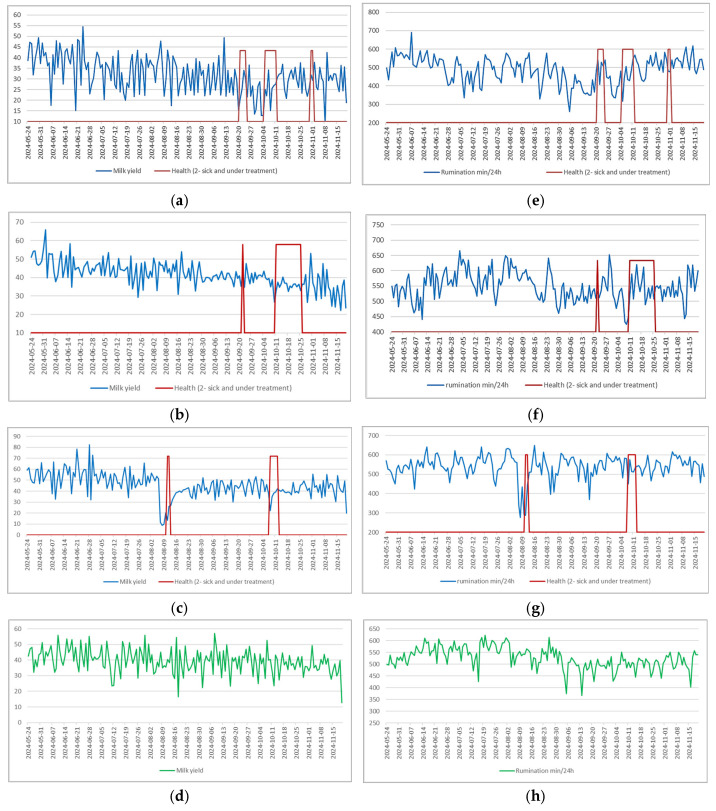
Health parameters over time. (**a**–**c**) milk yield data over time for three cows that experienced multiple sickness episodes and received medical treatment. Rumination (min per 24 h) for the same three cows (**e**–**g**) and corresponding data for a healthy cow: milk yield in (**d**), and rumination time in (**h**).

The absence of strong Pearson correlations indicates that the relationship between health parameters and disease status is neither instantaneous nor strictly linear, particularly at the individual cow level. Pearson correlation assesses pointwise linear associations and is therefore sensitive to measurement noise, temporal misalignment, and short-term variability, which are inherent characteristics of biological time-series data. To better understand the temporal dynamics of health-related parameters, the raw time-series data were first visualized in an exploratory analysis. Plotting the most informative parameters enables qualitative assessment of how these variables evolve and how they respond to health disturbances. Figure 10 presents the variation curves of selected parameters, with red segments indicating periods during which a veterinarian clinically confirmed illness. The blue line in each plot shows the actual daily values of the health parameter while the red line indicates the disease status: 2 denotes that the cow was sick and treated with medication, and 0 denotes a healthy cow.

Visual inspection shows more pronounced changes in milk yield and rumination time are observed for the third cow during clinically confirmed illness periods (Figure 10c,d). In contrast, for the first and second sick cows, similar fluctuations in these parameters are also present outside the periods labeled as illness, indicating that such changes are not uniquely associated with clinically identified disease events. It is clear that the relevance and sensitivity of individual parameters vary with the type of illness and each cow’s specific physiological response. In addition, production-related parameters differ substantially between individual cows, reflecting differences in lactation stage, genetics, productivity potential, and management conditions. As a result, even basic statistical measures, such as means or standard deviations, vary considerably across animals.

These observations provide intuitive insight into parameter behavior and motivate subsequent quantitative analysis using temporally smoothed representations, such as moving averages. In this study, a causal one-week (7-day) moving-average window was used, such that each smoothed value at day t was computed using only the current day and the preceding six days (no future observations were included). The application of moving averages is statistically justified, as temporal smoothing reduces high-frequency variance, increases the signal-to-noise ratio, and enhances the detectability of low-amplitude, persistent trends. This transformation enables more reliable assessment of time-dependent associations between health indicators and disease onset that are not captured by raw-value correlation analysis:

(2) $${MA}_{t}=\frac{1}{w}\;\sum_{i=0}^{w-1}y_{t-i},$$

where $\;{MA}_{t}$—Moving average for t element, w—window size, $y_{t}$—data instance.

**Figure 11:**
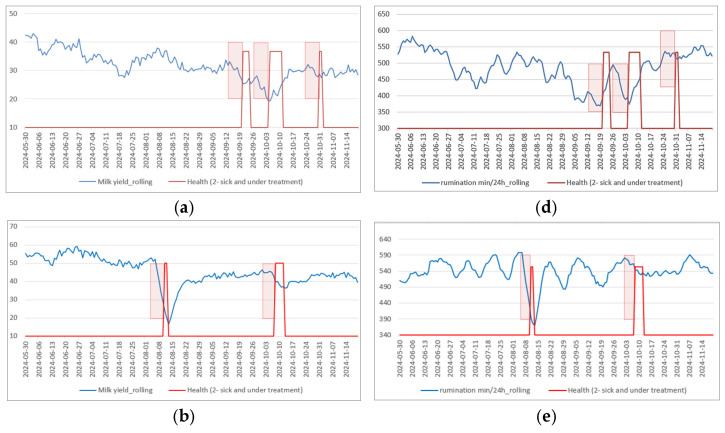

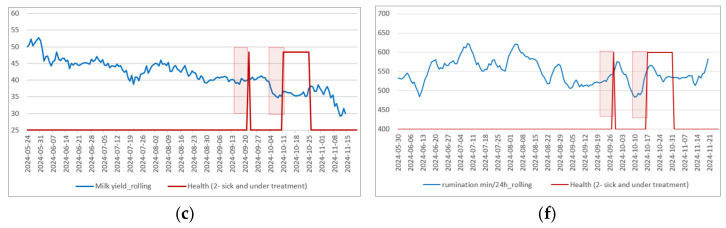
Health parameters vary in time. Milk yield parameters of 3 cows with a few sicknesses and treatments with medications ~1 week period before the treatment. (**a**) Time-series analysis of milk yield for Cow 1; (**b**) Time-series analysis of milk yield for Cow 2; (**c**) Time-series analysis of milk yield for Cow 3; (**d**) Time-series analysis of rumination for Cow 1; (**e**) Time-series analysis of rumination for Cow 2; (**f**) Time-series analysis of rumination for Cow 3.

Figure 11 illustrates rolling-average time series of milk yield and rumination time for three cows that experienced clinically confirmed illness episodes, together with corresponding veterinary health annotations (highlighted in red). Temporal smoothing reduces short-term variability and reveals broader trends; however, the responses of individual cows remain highly heterogeneous. For the first cow (Figure 11a,d), and the second cow (Figure 11b,e), visible and temporally aligned reductions in both milk yield and rumination are observed during illness periods. In contrast, the third cow (Figure 11c,f) exhibits similar fluctuations both during and outside clinically confirmed sick intervals, making these changes insufficiently distinctive as standalone indicators.

These observations highlight the limitations of relying on a single type of parameter for health assessment, as well as the impact of infrequent veterinary examinations and insufficiently detailed clinical records.

### 5.2. Visual Data Analysis

To evaluate the performance of the proposed model, several alternative architectures commonly used in image-based classification tasks (U-Net, O-Net, and ResNet) were implemented as baseline models. For segmentation-based architectures (U-Net and O-Net), the decoder output was replaced with a global average pooling layer followed by a fully connected classification head to enable image-level classification.

All models were trained and evaluated using the same thermal image input resolution, identical data augmentation strategies, and the same cow-level train/validation/test split after image augmentation. In addition, all models were trained under the same optimization settings, using the Adam optimizer with a learning rate of 0.0001, a batch size of 16, and 160 training epochs, with dropout applied for regularization, ensuring an equivalent training budget and a fair comparison. All experiments were implemented using PyTorch (version 2.4.1) and executed on a GPU-enabled workstation.

**Figure 12:**
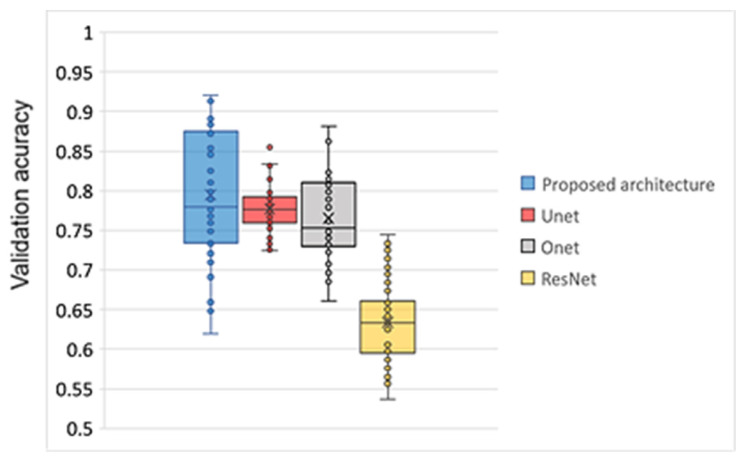
Comparison of the proposed model with existing architectures.

Figure 12 illustrates the distribution of validation accuracy values obtained across training epochs for the evaluated model architectures. The proposed architecture consistently achieves the highest validation accuracy throughout training, exhibiting both the highest median value and the highest upper range among all compared models. Validation accuracy for the proposed model frequently exceeds 0.85 and approaches 0.9 during later epochs, with a maximum of 0.92, indicating strong convergence behavior and stable generalization performance.

The U-Net and O-Net architectures show comparable validation accuracy trajectories across epochs, with median values predominantly in the range of approximately 0.75–0.80. U-Net demonstrates slightly more stable performance, as evidenced by a narrower interquartile range, whereas O-Net displays greater variability across epochs. Nevertheless, both architectures remain consistently below the proposed model’s performance throughout training. In contrast, the ResNet50 architecture yields substantially lower validation accuracy over epochs, with median values around 0.63–0.65 and limited variation. This suggests that ResNet50 converges to a suboptimal solution for the considered task and fails to achieve competitive validation performance relative to the other architectures.

**Figure 13:**
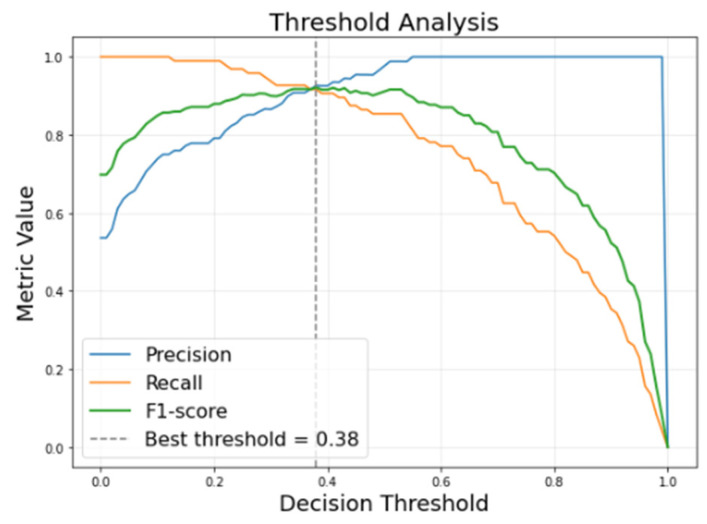
Threshold selection analysis.

To convert probabilistic model outputs into binary class predictions, a decision threshold analysis was performed. Figure 13 illustrates the variation in precision, recall, and F1-score as a function of the classification threshold. The optimal operating threshold was selected by maximizing the F1-score, resulting in a threshold value of 0.38. This threshold provides a balanced trade-off between precision and recall while maintaining a low false-positive rate, which is critical for practical deployment in dairy farm environments. The selected threshold was applied consistently across the test set and used for all reported confusion matrices and evaluation metrics.

**Figure 14:**
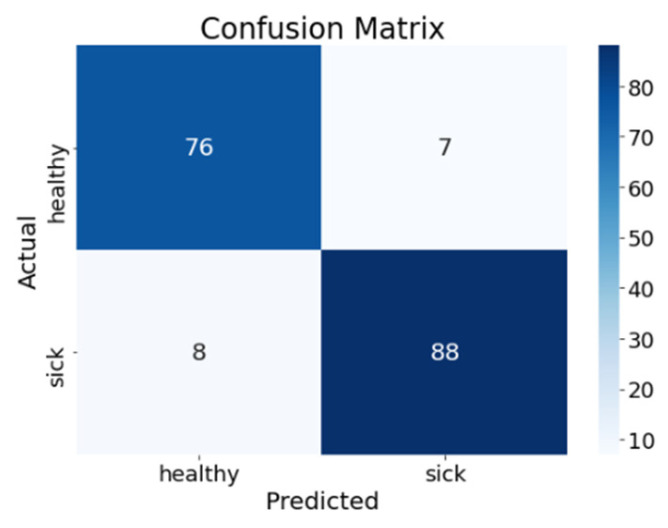
Confusion matrix of the proposed model.

The confusion matrix (Figure 14) illustrates the prediction performance of the final classification model, comparing predicted class labels against veterinarian-verified ground truth. The top-left cell shows that 76 healthy cows were correctly classified as healthy (true negatives), and the bottom-right cell shows that 88 sick cows were correctly classified as sick (true positives). The model produced 7 false positives, corresponding to healthy cows that were incorrectly classified as sick, and 8 false negatives, representing cases in which cows diagnosed as sick by a veterinarian were classified as healthy by the model. From a practical perspective, the low number of false positives suggests that the system maintains relatively conservative alerting behavior, limiting unnecessary treatment interventions and associated labor and animal stress. The false-negative cases likely correspond to early-stage or subtle infections in which thermal or sensor-based deviations were not yet sufficiently pronounced for confident detection. These results highlight the trade-off between sensitivity and specificity and underscore the importance of threshold selection in balancing missed detections against false alerts in real-world farm deployment.

**Figure 15:**
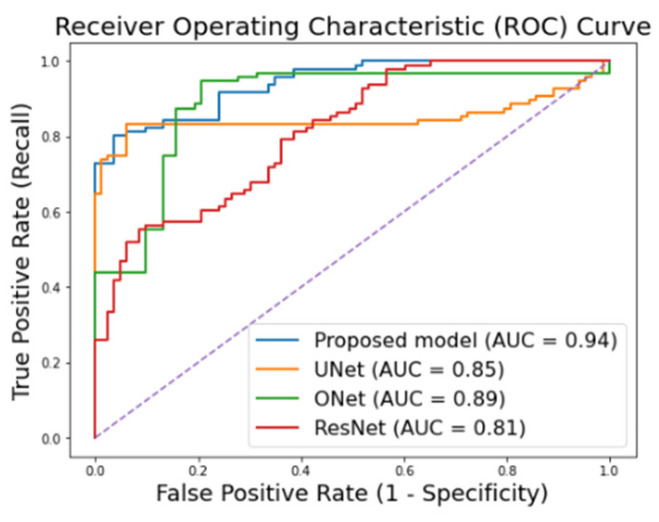
Proposed and other deep learning models’ ROC curves.

Figure 15 presents the receiver operating characteristic (ROC) curves comparing the proposed model with several baseline deep learning architectures, including U-Net, O-Net, and ResNet. The proposed model achieves the highest area under the ROC curve (AUC = 0.94), indicating superior discriminative capability in distinguishing between healthy and sick cows compared with U-Net (AUC = 0.85), O-Net (AUC = 0.89), and ResNet (AUC = 0.81).

Aggregating production parameters with thermal images improved overall classification accuracy and enhanced the model’s ability to reliably distinguish healthy from sick cows (see Table 4). Based on the results, aggregating production parameters with thermal images improved classification performance. Healthy cows’ F1-score increased from 0.91 to 0.93, corresponding to an improvement of ~2%. Sick cows F1-score increased from 0.92 to 0.95, corresponding to an improvement of ~3%. Recall for sick cows also improved slightly, from 0.92 to 0.95, indicating a higher detection rate of infected animals. Precision values showed consistent gains across both classes when thermal images were included. Overall, the multimodal model combining production parameters, bolus sensor data, and thermal images improved classification performance by approximately 2–3%, with the most pronounced gains observed in the detection of sick cows.

## 6. Discussion

Additional observational insights were obtained regarding the relationship between cow facial expressions and health status, extending beyond previously published findings. Table 5 summarizes the *p*-values obtained from statistical analyses of facial expression features (eyes, ears, nose, and muzzle) across three health conditions: clinical mastitis, subclinical mastitis, and hoop disease. For each condition, 15 cows (in total 60) per group were included and statistically tested.

**Figure 16:**
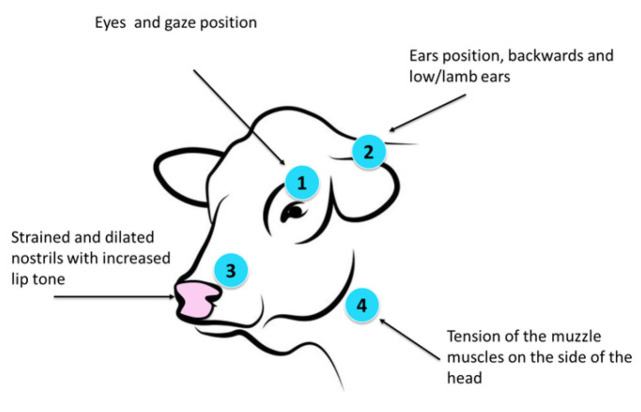
Schematic representation of facial regions used for observational analysis of cow facial expressions: (1) eye region (including gaze and pupil position), (2) ear position, (3) nose and nostrils, and (4) muzzle and lip muscle tension.

Clinically affected cows frequently exhibited partially closed or asymmetrical eyes, increased tension around the muzzle, and altered nostril appearance, features that were rarely observed in healthy individuals (see Figure 16). In contrast, cows diagnosed with subclinical mastitis showed facial expression scores comparable to those of healthy controls, with only subtle and inconsistent deviations. These observations highlight an important limitation of facial analysis: while it appears sensitive to clinically evident disease, its utility for detecting early-stage or mild conditions remains limited within the scope of this exploratory study. The statistical associations reported in Table 5 should therefore be interpreted with caution. The analysis was conducted on a relatively small sample, and no correction for multiple hypothesis testing was applied. As a result, the reported *p*-values are intended to provide preliminary, explanatory insight rather than confirmatory evidence of robust disease–facial feature relationships.

In this study, “hoof disease” refers to clinically diagnosed hoof-related locomotor disorders, including inflammatory or infectious claw conditions such as sole ulcers, digital dermatitis, and interdigital infections, which are among the most common causes of lameness in dairy cows. These disorders are associated with pain, altered gait, and reduced weight bearing, and therefore have a direct impact on animal welfare and behavioral and facial expression responses.

For cows with clinical mastitis, statistically significant differences were observed in the eye (p = 0.021) and nose (p = 0.039) features, whereas differences in ear position (p = 0.269) and muzzle features (p = 0.118) were not statistically significant, indicating clear changes in facial expression associated with clinically apparent disease. Similarly, in cases of hoof disease, significant differences were detected for the eyes (p = 0.013), nose (p = 0.034) suggesting that these facial regions are sensitive indicators of pain or discomfort related to locomotor disorders. However, ear and muzzle position did not show statistically significant differences across any of the examined conditions (p > 0.05), implying limited discriminatory power of this feature in the present dataset.

Observations further indicate substantial inter-individual variability within disease groups. Some clinically affected cows showed pronounced facial changes, while others exhibited only minor alterations. This variability suggests that facial expression may be influenced not only by disease presence but also by individual-specific and context-dependent factors, as indicated by the heterogeneous facial responses observed among cows diagnosed with the same condition. These findings should be regarded as preliminary and hypothesis-generating. Validation on larger datasets, ideally across multiple farms and disease stages and with appropriate multiple-testing correction, is required to determine the robustness and generalizability of facial expression–based indicators. Analysis over time may further clarify whether facial changes precede clinical diagnosis and could therefore contribute to early-warning systems.

To enable controlled acquisition of breath data, the MQ138 sensor unit was installed within an enclosed feeding station. However, the physical constraints of the feeding station introduced a critical limitation related to gas retention. As a result, the findings associated with breath-based acetone sensing should be considered preliminary engineering observations, rather than validated diagnostic outcomes. While the sensor technology itself is viable, future implementations should prioritize open-air sampling configurations combined with controlled ventilation, directional airflow channels, or active air purging mechanisms to minimize residual gas accumulation. In addition, alternative sensing strategies—such as photoacoustic gas sensors or selective metal-oxide sensor arrays with faster response times—may improve specificity and robustness under farm conditions.

The application of the C1001 60 GHz mmWave radar for monitoring heart and respiratory rates showed potential for remote health assessment; however, these results likewise represent exploratory feasibility findings. The complex industrial environment of the milking robot introduced substantial signal interference. The sensor was positioned in close proximity to the milking robot to capitalize on the animal’s stationary position during milking. However, this environment is characterized by a high density of metallic structures and the dynamic movement of the robotic arm. These factors generated significant electromagnetic noise and background clutter, which adversely affected the signal-to-noise ratio and the reliability of the raw data.

Future technical optimizations may include sensor repositioning to reduce multipath reflections, adaptive beamforming to suppress background clutter, shielding or isolation of the radar module, and the use of multi-antenna configurations. On the signal-processing side, advanced motion-separation techniques, such as adaptive filtering, frequency-domain decomposition, or machine learning based denoising, could improve discrimination between physiological micro-movements and mechanical artifacts.

Furthermore, a critical limitation of this preliminary study was the absence of simultaneous validation against a reference standard. Consequently, while the initial results are promising, the gas-sensing and mmWave radar components are presented as proof-of-concept technologies, and rigorous validation against established contact-based methods (e.g., ECG or wearable pulse oximeters) will be required before practical farm deployment.

Model interpretability is an important consideration for the practical adoption of AI-based decision support systems in livestock management. In this study, feature relevance was assessed during data preparation using mathematical and statistical criteria to identify informative numerical parameters prior to model training. This analysis guided feature selection and aggregation but did not provide explanations of individual model predictions. Interpreting the internal decision mechanisms of deep multimodal architectures remains challenging, particularly when combining numerical and image-based inputs. Future work should therefore incorporate dedicated interpretability techniques applied after model training, such as feature attribution methods for numerical data and visualization approaches (e.g., saliency maps or Grad-CAM) for thermal images, to better understand which parameters and image regions contribute most to disease prediction. Such analyses would enhance transparency, support biological plausibility, and facilitate practical deployment in farm decision-support settings.

The proposed multimodal framework was intentionally designed to build on infrastructure that already exists in many modern dairy farms, such as automated milking systems, intraruminal bolus sensors, and camera-based monitoring. As a result, the incremental deployment cost is mainly associated with the installation of thermal cameras and the software layer required for data fusion and AI-based analysis, rather than with large-scale hardware replacement.

From an operational perspective, all sensors were integrated into routine farm workflows without interfering with milking or animal handling, demonstrating that the system can operate continuously in a real commercial environment. The software architecture is vendor-agnostic and relies on standard data exports and identifiers (e.g., RFID and milking session IDs), allowing straightforward integration with existing herd management platforms such as DeLaval DelPro and, in principle, with other widely used systems. This design supports scalability and facilitates future multi-farm and multi-platform deployment. In practical deployment, the system can be used to flag cows exhibiting sustained deviations from individual baselines, prompting targeted veterinary inspection rather than herd-level intervention, thereby supporting more efficient health monitoring and decision-making.

## 7. Conclusions

This study demonstrates the effectiveness of a multimodal AI framework for health disorder detection in dairy cows under commercial farm conditions. By integrating production data, intra-ruminal physiological measurements, and non-contact thermal imaging, the proposed system provides a more reliable health assessment than single-modality approaches. The multimodal model achieved an overall accuracy of 91.62% and an AUC of 0.94, indicating strong discriminative capability. The recognition rate for sick cows was 0.92–0.95, and integrating production parameters with thermal images improved classification performance by approximately 2–3%, increasing the F1 score for sick cows from 0.92 to 0.95. Correlation analysis did not show strong linear relationships between individual parameters and disease status, confirming the need for multimodal, nonlinear modeling.

Overall, the results demonstrate that multimodal AI-driven sensing can improve discrimination between healthy and infected cows and reduce false alarms in retrospective analysis. However, some early-stage or subtle infections were not detected, indicating that prospective early disease detection remains a direction for future validation.

## 8. Patents

Reg. No. K158-137 LT Invention “Non-contact system and method for monitoring the health status of cows”.

## Figures and Tables

**Table 1:** Data sampling frequencies.

Data Source	Time Span	Sampling Frequency
Veterinary treatments	From birth to 20 October 2025	Recorded per clinical event
Physiological & production	1 January 2025–20 October 2025	Every milking (~2–3× daily)
Thermal imaging	5 consecutive days	3 images/milking session
Bolus sensor: temperature, activity, rumination	1 January 2025–20 October 2025	Every 10 min
Bolus sensor: water intake	1 January 2025–20 October 2025	Once per 24 h

**Table 2:** Structure of the collected numerical-type dataset.

Name of Numerical Parameter	Explanation of the Parameter
Temperature	Cows’ temperature
Normal temperature	Cows’ stomach temperature
Rumination (min/24 h)	How many minutes did the cow ruminate during the day
Is sick	0—healthy cow, 1—possibly sick cow, 2—sick cow
Water intake (L)	Litre of water drunk by the cow during a day
Milk yield (kg)	Milk that the cow produced in kilograms during a day
Milk yield (kg) LF, RF, LB, RB	Milk yield (kg) per udder quarter (LF, RF, LB, RB), representing milk production from the left front, right front, left back, and right back quarters
Max milk flow (kg/min) LF, RF, LB, RB	Maximum daily milk flow from each udder quarter (LF, RF, LB, RB), corresponding to the left front, right front, left back, and right back quarters.
Avg. milk flow (kg/min) LF, RF, LB, RB	Average daily milk flow per udder quarter (LF, RF, LB, RB), representing the left front, right front, left back, and right back quarters.

**Table 3:** Specifications of the thermal camera.

Spectral Range	8~14 μm
Resolution	256 × 192 Pixels
Frame rate	25 Hz
Object temperature range	−20~150 °C (−4~302 °F), 150 °C~550 °C (302~1022 °F)
Accuracy	Temperature Accuracy: ±3.6 °F (2 °C) Temperature Resolution: down to 0.1 °C (0.1 °F). Note: The potential thermal error will be less than ±1.8 °F (1 °C) when the target temperature is below 212 °F (100 °C).

**Table 4:** Evaluation metrics of the proposed model.

	Thermal Images		Production Parameters + Bolus Sensor + Thermal Images	
	Healthy	Sick	Healthy	Sick
Precision	0.90	0.93	0.91	0.94
Recall	0.92	0.92	0.94	0.95
F1-score	0.91	0.92	0.93	0.95

**Table 5:** *p*-values of conducted observations.

	Clinical Mastitis	Subclinical Mastitis	Hoof Disease
Eyes	0.021	0.015	0.013
Ears	0.269	0.198	0.062
Nose	0.039	0.172	0.034
Muzzle	0.118	0.178	0.146

## Data Availability

The data presented in this study are available on request from the corresponding author.

## References

[1] Ezanno P., Picault S., Beaunée G., Bailly X., Muñoz F., Duboz R., Monod H., Guégan J.-F. (2021). Research Perspectives on Animal Health in the Era of Artificial Intelligence. Vet. Res..

[2] Zhang L., Guo W., Lv C., Guo M., Yang M., Fu Q., Liu X. (2024). Advancements in Artificial Intelligence Technology for Improving Animal Welfare: Current Applications and Research Progress. Anim. Res. One Health.

[3] Sguizzato A.L.L., da Silva T.E., Chagas J.C.C., Argüelo A.M., Gonçalves N.M., Marcondes M.I. (2024). Understanding the Dynamics of Mastitis in Milk Yield: Decoding Onset and Recovery Patterns in Response to Mastitis Occurrence. JDS Commun..

[4] Kavlak A.T., Pastell M., Uimari P. (2023). Disease Detection in Pigs Based on Feeding Behaviour Traits Using Machine Learning. Biosyst. Eng..

[5] Wang H., Zhang X., Meng X., Song W., Chen Z. (2023). Electronic Sheepdog: A Novel Method in with UAV-Assisted Wearable Grazing Monitoring. IEEE Internet Things J..

[6] Elhorst R., Syposz M., Wojczulanis-Jakubas K. (2025). BEHAVE—Facilitating Behaviour Coding from Videos with AI-Detected Animals. Ecol. Inform..

[7] Manikandan V., Neethirajan S., Manikandan V., Neethirajan S. (2025). AI-Powered Vocalization Analysis in Poultry: Systematic Review of Health, Behavior, and Welfare Monitoring. Sensors.

[8] Gomes C., Coheur L., Tilley P. (2025). A Review of Multimodal AI in Veterinary Diagnosis: Current Trends, Challenges, and Future Directions. IEEE Access.

[9] Liebe D.M., Steele N.M., Petersson-Wolfe C.S., De Vries A., White R.R. (2022). Practical Challenges and Potential Approaches to Predicting Low-Incidence Diseases on Farm Using Individual Cow Data: A Clinical Mastitis Example. J. Dairy Sci..

[10] He P., Chen Z., Yu H., Hayat K., He Y., Pan J., Lin H., He P., Chen Z., Yu H. (2022). Research Progress in the Early Warning of Chicken Diseases by Monitoring Clinical Symptoms. Appl. Sci..

[11] Gayathri S.L., Bhakat M., Mohanty T.K., Kumar R.R., Chaturvedi K.K., Kumar S. (2025). Advanced Mastitis Detection in *Bos indicus* Cows: A Deep Learning Approach Integrated with Infrared Thermography. J. Therm. Biol..

[12] Dhaliwal Y., Bi H., Neethirajan S. (2025). Bimodal Data Analysis for Early Detection of Lameness in Dairy Cows Using Artificial Intelligence. J. Agric. Food Res..

[13] Siachos N., Neary J.M., Smith R.F., Oikonomou G. (2024). Automated Dairy Cattle Lameness Detection Utilizing the Power of Artificial Intelligence; Current Status Quo and Future Research Opportunities. Vet. J..

[14] Rebez E.B., Sejian V., Silpa M.V., Kalaignazhal G., Thirunavukkarasu D., Devaraj C., Nikhil K.T., Ninan J., Sahoo A., Lacetera N. (2024). Applications of Artificial Intelligence for Heat Stress Management in Ruminant Livestock. Sensors.

[15] Cavallini D., Giammarco M., Buonaiuto G., Vignola G., De Matos Vettori J., Lamanna M., Prasinou P., Colleluori R., Formigoni A., Fusaro I. (2025). Two Years of Precision Livestock Management: Harnessing Ear Tag Device Behavioral Data for Pregnancy Detection in Free-Range Dairy Cattle on Silage/Hay-Mix Ration. Front. Anim. Sci..

[16] Nebel R.L., Dransfield M.G., Jobst S.M., Bame J.H. (2000). Automated Electronic Systems for the Detection of Oestrus and Timing of AI in Cattle. Anim. Reprod. Sci..

[17] Michelena Á., Fontenla-Romero Ó., Luis Calvo-Rolle J. (2025). A Review and Future Trends of Precision Livestock over Dairy and Beef Cow Cattle with Artificial Intelligence. Log. J. IGPL.

[18] Liu N., Qi J., An X., Wang Y., Liu N., Qi J., An X., Wang Y. (2023). A Review on Information Technologies Applicable to Precision Dairy Farming: Focus on Behavior, Health Monitoring, and the Precise Feeding of Dairy Cows. Agriculture.

[19] Bobbo T., Biffani S., Taccioli C., Penasa M., Cassandro M. (2021). Comparison of Machine Learning Methods to Predict Udder Health Status Based on Somatic Cell Counts in Dairy Cows. Sci. Rep..

[20] Neethirajan S. (2023). Artificial Intelligence and Sensor Technologies in Dairy Livestock Export: Charting a Digital Transformation. Sensors.

[21] Shi Z., Chang F., Jia Y., Li J., Qiu Y., Miao J., Jiang W., Guo X., Han X., Tang W. (2024). Classifying and Understanding of Dairy Cattle Health Using Wearable Inertial Sensors with Random Forest and Explainable Artificial Intelligence. IEEE Sens. Lett..

[22] Lee M., Seo S., Lee M., Seo S. (2021). Wearable Wireless Biosensor Technology for Monitoring Cattle: A Review. Animals.

[23] Hajnal É., Kovács L., Vakulya G., Hajnal É., Kovács L., Vakulya G. (2022). Dairy Cattle Rumen Bolus Developments with Special Regard to the Applicable Artificial Intelligence (AI) Methods. Sensors.

[24] Leliveld L.M.C., Brandolese C., Grotto M., Marinucci A., Fossati N., Lovarelli D., Riva E., Provolo G. (2024). Real-Time Automatic Integrated Monitoring of Barn Environment and Dairy Cattle Behaviour: Technical Implementation and Evaluation on Three Commercial Farms. Comput. Electron. Agric..

[25] Lewis Baida B.E., Swinbourne A.M., Barwick J., Leu S.T., van Wettere W.H.E.J. (2021). Technologies for the Automated Collection of Heat Stress Data in Sheep. Anim. Biotelemetry.

[26] Pil-Kee M., Kazuyuki M., Tae H.K. (2024). The Evolving Landscape of Artificial Intelligence Applications in Animal Health. Indian J. Anim. Res..

[27] Casas R., Hermosa A., Marco Á., Blanco T., Zarazaga-Soria F.J., Casas R., Hermosa A., Marco Á., Blanco T., Zarazaga-Soria F.J. (2021). Real-Time Extensive Livestock Monitoring Using LPWAN Smart Wearable and Infrastructure. Appl. Sci..

[28] Hunter L.B., Baten A., Haskell M.J., Langford F.M., O’Connor C., Webster J.R., Stafford K. (2021). Machine Learning Prediction of Sleep Stages in Dairy Cows from Heart Rate and Muscle Activity Measures. Sci. Rep..

[29] Swain S., Pattnayak B.K., Mohanty M.N., Jayasingh S.K., Patra K.J., Panda C. (2024). Smart Livestock Management: Integrating IoT for Cattle Health Diagnosis and Disease Prediction Through Machine Learning. Indones. J. Electr. Eng. Comput. Sci..

[30] Zhang Y., Zhang Y., Jiang H., Du H., Xue A., Shen W. (2024). New Method for Modeling Digital Twin Behavior Perception of Cows: Cow Daily Behavior Recognition Based on Multimodal Data. Comput. Electron. Agric..

[31] Mg W.H.E., Zin T.T., Tin P., Aikawa M., Honkawa K., Horii Y. (2025). Automated Cattle Monitoring System for Calving Time Prediction Using Trajectory Data Embedded Time Series Analysis. IEEE Open J. Ind. Electron. Soc..

[32] Zhang S., Sailunaz K., Neethirajan S., Zhang S., Sailunaz K., Neethirajan S. (2025). Micro-Expression-Based Facial Analysis for Automated Pain Recognition in Dairy Cattle: An Early-Stage Evaluation. AI.

[33] Patel S., Neethirajan S. (2025). CowPain Check: AI-Based Facial Expression Analysis for Dairy Cow Welfare. TechRxiv.

[34] Chu M., Si Y., Li Z., Li Q., Liu G. (2025). Multi-Feature Image Layers Fusion for Accurate Detection of Dairy Cow Mastitis Using Deep Learning. Comput. Electron. Agric..

[35] Khan M.F., Thorup V.M., Luo Z. (2024). Delineating Mastitis Cases in Dairy Cows: Development of an IoT-Enabled Intelligent Decision Support System for Dairy Farms. IEEE Trans. Ind. Inform..

[36] Pakrashi A., Ryan C., Guéret C., Berry D.P., Corcoran M., Keane M.T., Namee B.M. (2023). Early Detection of Subclinical Mastitis in Lactating Dairy Cows Using Cow-Level Features. J. Dairy Sci..

[37] Grodkowski G., Szwaczkowski T., Koszela K., Mueller W., Tomaszyk K., Baars T., Sakowski T. (2022). Early Detection of Mastitis in Cows Using the System Based on 3D Motions Detectors. Sci. Rep..

[38] Zhang Q., Yang Y., Liu G., Ning Y., Li J., Zhang Q., Yang Y., Liu G., Ning Y., Li J. (2023). Dairy Cow Mastitis Detection by Thermal Infrared Images Based on CLE-UNet. Animals.

[39] Wang Y., Kang X., He Z., Feng Y., Liu G. (2022). Accurate Detection of Dairy Cow Mastitis with Deep Learning Technology: A New and Comprehensive Detection Method Based on Infrared Thermal Images. Animal.

[40] da Silva R.A.B., Pandorfi H., Cordeiro F.R., Soares R.G.F., de Medeiros V.W.C., de Almeida G.L.P., Barbosa Filho J.A.D., Marinho G.T.B., da Silva M.V. (2024). A New Way to Identify Mastitis in Cows Using Artificial Intelligence. AgriEngineering.

[41] Batista R., Stošic B., Pandorfi H., Almeida G., Silva M., Batista P. (2021). Classification of Thermal Images of Bovine Mastitis by Computer Vision. Int. J. Comput. Appl..

[42] Urban-Chmiel R., Mudroň P., Abramowicz B., Kurek Ł., Stachura R., Urban-Chmiel R., Mudroň P., Abramowicz B., Kurek Ł., Stachura R. (2024). Lameness in Cattle—Etiopathogenesis, Prevention and Treatment. Animals.

[43] Kleinhenz M.D., Viscardi A.V., Coetzee J.F. (2021). Invited Review: On-Farm Pain Management of Food Production Animals. Appl. Anim. Sci..

[44] Brunt M.W., Ritter C., Renaud D.L., LeBlanc S.J., Kelton D.F. (2025). Dairy Producers’ Awareness, Perceptions, and Barriers to Early Detection and Treatment of Lameness on Dairy Farms: A Qualitative Focus Group Study. J. Dairy Sci..

[45] Nejati A., Bradtmueller A., Shepley E., Vasseur E. (2023). Technology Applications in Bovine Gait Analysis: A Scoping Review. PLoS ONE.

[46] Shen Y., Li B., Wang Y., Li Q., Zhang Z. (2025). An Algorithm for Detecting Cow Lameness Based on Ensemble Learning of Keypoint Motion Features. J. Dairy Sci..

[47] Barney S., Dlay S., Crowe A., Kyriazakis I., Leach M. (2023). Deep Learning Pose Estimation for Multi-Cattle Lameness Detection. Sci. Rep..

[48] Leclercq A., Ask K., Mellbin Y., Byström A., Söderlind M., Telezhenko E., Bergsten C., Haubro Andersen P., Rhodin M., Hernlund E. (2025). Kinematic Changes in Dairy Cows with Induced, Unilateral Forelimb Lameness During Straight Line Walk. Animal.

[49] Bradtmueller A., Nejati A., Shepley E., Vasseur E., Bradtmueller A., Nejati A., Shepley E., Vasseur E. (2023). Applications of Technology to Record Locomotion Measurements in Dairy Cows: A Systematic Review. Animals.

[50] Christodoulopoulos G. (2025). Subacute Ruminal Acidosis in Cattle: A Critical Review of Clinical Management. Vet. Res. Commun..

[51] Arshad J., Siddiqui T.A., Sheikh M.I., Waseem M.S., Nawaz M.A.B., Eldin E.T., Rehman A.U. (2023). Deployment of an Intelligent and Secure Cattle Health Monitoring System. Egypt. Inform. J..

[52] Zhang K., Zhang Z., Li Z., Qiao Y. (2016). Joint Face Detection and Alignment Using Multi-Task Cascaded Convolutional Networks. IEEE Signal Process. Lett..

[53] Zhao H., Shi J., Qi X., Wang X., Jia J. (2017). Pyramid Scene Parsing Network. arXiv.

[54] He K., Zhang X., Ren S., Sun J. (2015). Deep Residual Learning for Image Recognition. arXiv.

